# The Effects of Rapamycin on the Proliferation, Migration, and Apoptosis of Human Tracheal Fibroblasts (HTrF) and Human Tracheal Epithelial Cells (HTEpiC)

**DOI:** 10.3390/jcm11030608

**Published:** 2022-01-25

**Authors:** Yan Liu, Jie Zhang, Jianhai Long, Xiaojian Qiu, Ting Wang, Juan Wang

**Affiliations:** Department of Respiratory, Beijing Tiantan Hospital, Capital Medical University, Beijing 100070, China; Liuyan12252021@163.com (Y.L.); jianhai8561729@sina.com (J.L.); 13718877685@163.com (X.Q.); wangtingttyy@163.com (T.W.); easy6365@163.com (J.W.)

**Keywords:** restenosis, airway stent, drug-eluting stent, human tracheal fibroblasts (HTrF), human tracheal epithelial cells (HTEpiC), rapamycin

## Abstract

Background: Restenosis after airway stenting needs to be addressed urgently. Rapamycin has been proven to inhibit restenosis elsewhere. This study aimed at observing its effects on the respiratory tract. Methods: CCK-8, wound healing, Transwell and apoptosis assays were performed to detect the effects of rapamycin on the survival, migration, and apoptosis, respectively, of human tracheal fibroblasts (HTrF) and human tracheal epithelial cells (HTEpiC). Results: The effective concentrations of paclitaxel, mitomycin C and rapamycin on HTrF were 10^−^^7^–10^−^^4^ mol/L, 10^−^^6^–10^−^^4^ mol/L, and 10^−^^5^–10^−^^4^ mol/L, respectively. At the effective concentrations, the inhibition rates of paclitaxel on HTEpiC were (43.03 ± 1.12)%, (49.49 ± 0.86)%, (55.22 ± 1.43)%, and (93.19 ± 0.45)%; the inhibition rates of mitomycin C on HTEpiC were (88.11 ± 0.69)%, (93.82 ± 0.96)%, and (94.94 ± 0.54)%; the inhibition rates of rapamycin on HTEpiC were (10.19 ± 0.35)% and (94.55 ± 0.71)%. At the concentration of (1–4) × 10^−^^5^ mol/L, the inhibition rate of rapamycin on HTrF was more than 50%, and that on HTEpiC was less than 20% (*p* < 0.05). Conclusions: Compared to paclitaxel and mitomycin C, rapamycin had the least effect on HTEpiC while effectively inhibiting HTrF. The optimum concentration range was (1–4) × 10^−5^ mol/L.

## 1. Introduction

The treatment of benign cicatricial airway stenosis (BCAS) has always been a difficult problem in the field of respiratory intervention medicine. For patients with BCAS that cannot be treated with conventional interventional therapy and surgery, airway stent implantation is the only option. Although airway stents have the advantage of being minimally invasive and timely airway management is possible, the restenosis rate is about 10~50% [1,2], which seriously affects the curative effect and prognosis of patients [3,4].

Previous studies have shown that the main causes of restenosis after stenting are: (1) the destruction of airway mucosa by airway lesions, (2) the injury to the airway mucosa by interventional therapy [5], and (3) the continuous irritation of the airway mucosa by the airway stent. These factors activate the inflammation, promote the excessive proliferation of fibroblasts and the formation of cicatricial tissue, and finally lead to restenosis [5,6,7,8]. A few studies have shown that in addition to fibroblasts, the progress of airway injury repair also needs the collaborative participation of epithelial cells. Intact epithelium could inhibit inflammation and promote apoptosis in fibroblasts. Therefore, complete epithelialization of the airway is also key to preventing restenosis after stenting [9,10,11,12,13].

Drug-eluting stents may solve the problem of airway restenosis. Paclitaxel [14,15,16] and mitomycin C [17,18,19]—eluting stents have been proven to effectively inhibit fibr006Fblast proliferation in vitro and in animal models, but they also inhibited epithelial cell proliferation [5,20,21,22,23]. Therefore, to solve restenosis after stenting, a drug that not only inhibits the proliferation of fibroblasts but also has little effect on the epithelialization of the airway needs to be identified.

Rapamycin is a macrolide antibiotic with an immunosuppressive effect. It has the following characteristics: (1) a larger safe dose range, (2) it selectively inhibits the proliferating cells, and (3) initiates cell cycle arrest by inhibiting the key enzyme (rapamycin target enzyme, TOR) that promotes cell proliferation, the entry of cells into the S phase by binding to intracellular receptor proteins, and the return of the cells from the G1 phase to the dormant phase or G0 phase. Based on the above characteristics, theoretically, rapamycin is an ideal drug for airway stent coating. Rapamycin has been proven to effectively inhibit restenosis in the cardiovascular system, vertebral artery, and urinary system [24,25,26]. However, whether it can be applied in airway stenting and its effect on inhibiting restenosis are still controversial [27,28].

To screen the antiproliferative drugs that have the least effect on complete epithelialization and provide a direction for solving the problem of restenosis after airway stenting, human tracheal fibroblasts (HTrF) and human tracheal epithelial cells (HTEpiC) were used to observe the effects of rapamycin on proliferation, migration, and apoptosis and were compared to those of paclitaxel and mitomycin C.

## 2. Materials and Methods

### 2.1. Materials

Cells: Human Tracheal Fibroblasts (HTrF, ScienCell, Carlsbad, CA, USA), and Human Tracheal Epithelial Cells (HTEpiC, ScienCell). The HTrF HTEpiC cell lines were provided by Shanghai Zhong Qiao Xin Zhou Biotechnology Co. Ltd. (Shanghai, China).

Drugs: Rapamycin (Solarbio, Beijing, China), paclitaxel (Solarbio), and mitomycin C (Solarbio).

### 2.2. Methods

Cell culture: HTrF were cultured in Dulbecco’s modified Eagle’s medium (DMEM, Gibco, Grand Island, NY, USA) containing 10% Fetal Bovine Serum (FBS, Gibco), and HTEpiC were cultured in Bronchial Epithelial Cell Medium (BEpiCM, ScienCell) containing 1% Bronchial Epithelial Cell Growth Stimulus (BEpiCGS, ScienCell). The cells were cultured in an incubator at 37 °C with 95% humidity and 5% CO_2_. The culture dish used for HTEpiC was coated with Poly-d-Lysine hydrobromide (PDL, Salarbio) at a concentration of 0.1 mg/mL for 2 h in advance and washed with PBS thrice before cells were seeded. The culture medium was changed daily.

Drug Preparation: (1) Paclitaxel, mitomycin C, and rapamycin powder were weighed and dissolved in Dimethyl Sulfoxide (DMSO, Solarbio, Beijing, China) to an initial concentration of 10^−3^ mol/L. The drugs were then diluted to 10^−4^, 10^−5^, 10^−6^, 10^−7^, 10^−8^, 10^−9^, 10^−10^, and 10^−11^ mol/L in complete medium. (2) Rapamycin solutions of different concentrations (1 × 10^−5^, 2 × 10^−5^, 4 × 10^−5^, 6 × 10^−5^, 8 × 10^−5^, and 10 × 10^−5^ mol/L) were prepared.

#### 2.2.1. CCK-8 Assay

The cell viability was detected using the cell counting kit-8 (CCK-8, BeiRen Chemical Science and Technology Ltd., Beijing, China). Cells at the logarithmic growth stage were seeded at 5000 cells/well with 100 µL medium in 96-well plates and routine-cultured in an incubator at 37 °C with 95% humidity and 5% CO_2_ for 24 h. The wells were divided into 6 rows and 10 columns. Column 1 was the blank group (containing culture medium), column 2 was the negative control group (containing cells and culture medium and DMSO, the concentration of DMSO was the same as that of the 10^−4^ drug concentration group), and columns 3 to 10 were the drug treatment groups (containing cells, culture medium, and drugs of different concentrations). Each group contained six wells. After 24 h, the original culture medium was discarded and 100 μL culture medium containing different concentrations of drugs was added to the corresponding group. The same amount of complete medium was added to the blank and negative control groups and the remaining marginal wells were filled with 0.9% normal saline. The 96-well plates were incubated for 24 h, 48 h, and 72 h. After incubation, the medium was removed and CCK-8 solution (10 µL CCK-8 in 100 µL culture medium) was added to each well and incubated for 4 h. The optical density (OD) was determined using a microplate reader at 450 nm. The mean OD value of the six wells was calculated, and each experiment was repeated three times. Inhibition rate = [(Ac − As)/(Ac − Ab)] × 100% (As, the absorbance of the drug treatment group; Ac, the absorbance of the negative control group; Ab, the absorbance of the blank group).

#### 2.2.2. Wound Healing Assay

Cells at the logarithmic growth stage were placed in a six-well plate with a cell density of 1 × 10^5^ cells/well and routine-cultured in an incubator at 37 °C with 95% humidity and 5% CO_2_ for 24 h. A 200-μL pipetting tip was used to vertically scratch the six-well plate to avoid tilting. The damaged or dead cells were washed away with PBS and then the 2 mL culture medium containing different concentrations of rapamycin was added to the treatment groups. The same amount of medium (culture medium and DMSO, the concentration of DMSO was the same as that of the 10^−4^ drug concentration group) was added to the negative control group. Photographs were taken at 0 and 24 h using a microscope and the wound healing area was calculated using ImageJ. The experiment was repeated thrice.

#### 2.2.3. Transwell Migration Assay

The capacity of the HTrF and HTEpiC to migrate was detected using Transwell chambers (8.0 µm pore size, 24-wells, Corning, Corning, NY, USA). A total of 1 × 10^4^ cells (100 µL) were placed in the upper chamber, and 700 μL medium containing 20% FBS was added to the lower chamber. The upper chamber was carefully immersed in the lower chamber liquid using sterile forceps and the cells were routine-cultured in the incubator at 37 °C with 95% humidity and 5% CO_2_ for 24 h. After 24 h, the chamber was fixed in 700 μL fixing solution (formaldehyde: acetone = 1:1) for 30 min. Then, the chamber was stained with 700 μL 0.1% crystal violet reagent for 15 min at room temperature. After the crystal violet staining, cells on the surface of the membrane were scraped out using a cotton swab. After that, the upper chambers were observed using an electron microscope and photographed to count the number of migrated cells. Each experiment was repeated three times.

#### 2.2.4. Flow Cytometric Analysis of Annexin V-FITC/7AAD Double Staining

The apoptosis rate was detected using the Annexin V-FITC/7AAD kit (BD Pharmingen, Franklin Lake, NJ, USA). Cells at the logarithmic growth stage were seeded at 1 × 10^5^ cells/well with 2 mL medium in six-well plates and routine-cultured in an incubator at 37 °C with 95% humidity and 5% CO_2_ for 24 h. Cells were trypsinized and centrifuged at 300× *g* at 4 °C for 5 min. Then, the cells were washed with 1 mL precooled PBS and centrifuged. The above process was repeated twice. The cells were resuspended in 200 μL binding buffer and 5 μL 7AAD was added. After mixing gently, the cells were kept at room temperature for 15 min in the dark. Then, the cells were filtered using a 200-mesh filter screen and flow cytometry was performed within 1 h to detect the cell apoptosis rate.

### 2.3. Statistical Analysis

SPSS 22.0 software was used for data analysis, and GraphPad Prism 8.0 (GraphPad Software, La Jolla, CA, USA) was used for graphing. The data were expressed as mean ± standard deviation (SD). All data were tested for normal distribution and homogeneity of variance, then analyzed using non-parametric tests (Kruskal–Wallis and Mann–Whitney). *p*-values < 0.05 were considered statistically significant.

## 3. Results

### 3.1. CCK-8 Assay

#### 3.1.1. The Effect of Drugs on the Proliferation of HTrF

**Figure 1:**
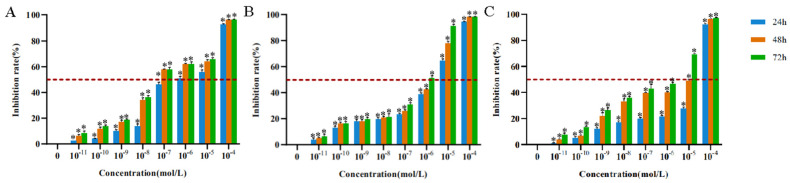
The inhibition of proliferation of HTrF at different concentrations of paclitaxel, mitomycin C, and rapamycin. * represents a statistically significant difference compared with the negative control group, *p* < 0.05. (**A**): The inhibition of proliferation of HTrF at different concentrations of paclitaxel. (**B**): The inhibition of proliferation of HTrF at different concentrations of mitomycin C. (**C**): The inhibition of proliferation of HTrF at different concentrations of rapamycin.

- Paclitaxel, mitomycin C, and rapamycin at different concentrations and action times could inhibit the proliferation of HTrF to varying degrees (Table 1).
- The effective concentration of paclitaxel with an inhibition rate of more than 50% on fibroblasts was 10^−7^–10^−4^ mol/L (Figure 1A).
- The effective concentration of mitomycin C with an inhibition rate of more than 50% on fibroblasts was 10^−6^–10^−4^ mol/L (Figure 1B).
- The effective concentration of rapamycin with an inhibition rate of more than 50% on fibroblasts was 10^−5^–10^−4^ mol/L (Figure 1C).

#### 3.1.2. The Effect of Drugs on the Proliferation of HTEpiC

**Figure 2:**
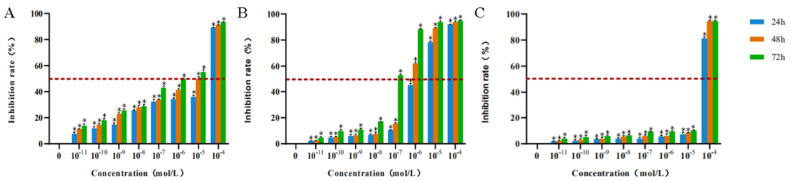
The inhibition of proliferation of HTEpiC at different concentrations of paclitaxel, mitomycin C, and rapamycin. * represents a statistically significant difference compared to the negative control group, *p* < 0.05. (**A**): The inhibition of proliferation of HTEpiC at different concentrations of paclitaxel. (**B**): The inhibition of proliferation of HTEpiC at different concentrations of mitomycin C. (**C**): The inhibition of proliferation of HTEpiC at different concentrations of rapamycin.

- Paclitaxel, mitomycin C, and rapamycin at different concentrations and action times could inhibit the proliferation of HTEpiC to varying degrees (Table 2).
- The 72 h inhibition rates of paclitaxel on HTEpiC at the effective concentration (10^−7^–10^−4^ mol/L) were (43.03 ± 1.12)%, (49.49 ± 0.86)%, (55.22 ± 1.43)%, and (93.19 ± 0.45)% (Figure 2A).
- The 72 h inhibition rates of mitomycin C on HTEpiC at the effective concentration (10^−6^–10^−4^ mol/L) were (88.11 ± 0.69)%, (93.82 ± 0.96)%, and (94.94 ± 0.54)% (Figure 2B).
- The 72 h inhibition rates of rapamycin on HTEpiC at the effective concentration (10^−5^–10^−4^ mol/L) were (10.19 ± 0.35)% and (94.55 ± 0.71)% (Figure 2C).

#### 3.1.3. Optimal Concentration Range of Rapamycin

**Figure 3:**
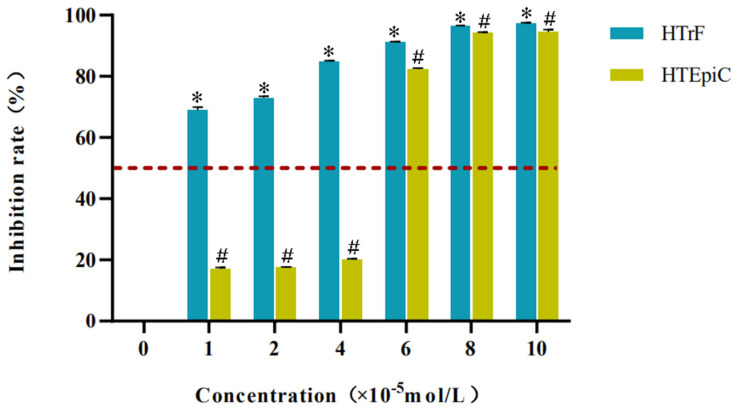
The inhibition rate of different concentrations of rapamycin on HTrF and HTEpiC. * represents a statistically significant difference compared to the negative control group (HTrF), *p* < 0.05; # represents a statistically significant difference compared to the negative control group (HTEpiC), *p* < 0.05.

In the concentration range of (1–4) × 10^−5^ mol/L, the inhibition rate of rapamycin on HTrF was more than 50%, and that on HTEpiC was less than 20% (*p* < 0.05). In the concentration range of (6–10) × 10^−5^ mol/L, the inhibition rate of rapamycin on HTrF and HTEpiC was more than 50% (*p* < 0.05) (Figure 3).

### 3.2. Wound Healing Assay

**Figure 4:**
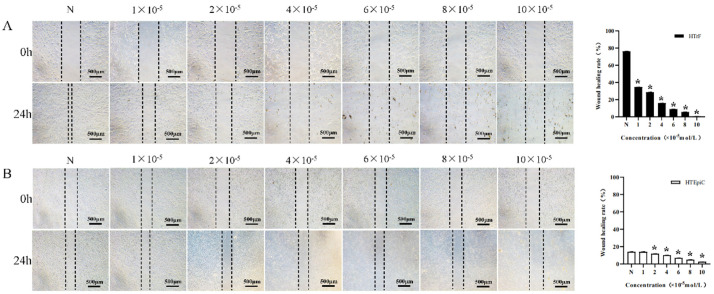
The effects of different concentrations of rapamycin on wound healing in HTrF and HTEpiC. * represents a statistically significant difference compared to the negative control group, *p* < 0.05. (**A**): Wound healing rate of HTrF with different concentrations of rapamycin. (**B**): Wound healing rate of HTEpiC with different concentrations of rapamycin.

The wound healing rates of the negative control and 1 × 10^−5^, 2 × 10^−5^, 4 × 10^−5^, 6 × 10^−5^, 8 × 10^−5^, and 10 × 10^−5^ mol/L rapamycin concentration groups on HTrF were (76.36 ± 0.11)%, (34.69 ± 0.08)%, (28.67 ± 0.18)%, (15.95 ± 0.18)%, (9.07 ± 0.03)%, (5.72 ± 0.13)%, and (0.61 ± 0.13)%, respectively. The wound healing rate of each group was lower than that of the negative control group, and the differences were statistically significant, *p* < 0.05 (Figure 4A).

The wound healing rates of the negative control and 1 × 10^−5^, 2 × 10^−5^, 4 × 10^−5^, 6 × 10^−5^, 8 × 10^−5^, and 10 × 10^−5^ mol/L rapamycin concentration groups on HTEpiC were (14.13 ± 0.12)%, (14.02 ± 0.16)%, (11.86 ± 0.11)%, (10.14 ± 0.19)%, (6.99 ± 0.15)%, (4.99 ± 0.13)%, and (2.57 ± 0.07)%, respectively. There was no significant difference in the healing rate between the negative control and 10^−5^ mol/L rapamycin concentration groups (*p* > 0.05). The wound healing rate of the other groups were lower than that of the negative control group, and the differences were statistically significant (*p* < 0.05) (Figure 4B).

### 3.3. Transwell Migration Assay

**Figure 5:**
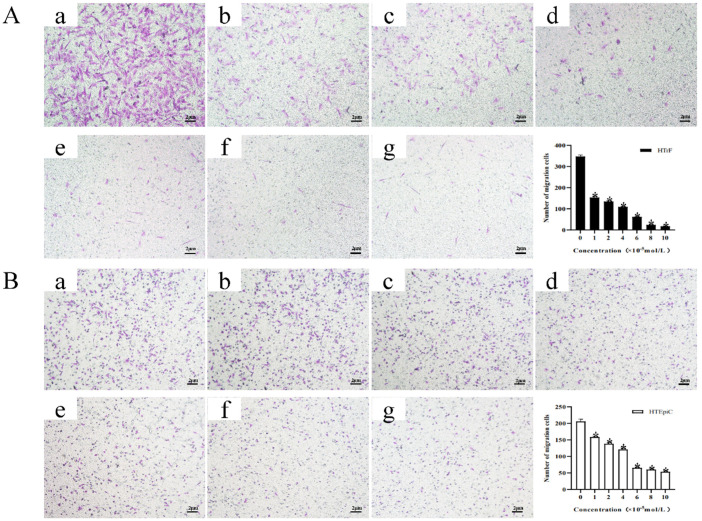
The effects of different concentrations of rapamycin on the migration ability of HTrF and HTEpiC. * represents a statistically significant difference compared to the negative control group, *p* < 0.05. (**A**-**a**): The number of migrated HTrF cells in the negative control group on HTrF, (**A**-**b**): The number of migrated HTrF cells in the 1 × 10^−5^ mol/L rapamycin group on HTrF. (**A**-**c**): The number of migrated HTrF cells in the 2 × 10^−5^ mol/L rapamycin group on HTrF. (**A**-**d**): The number of migrated HTrF cells in the 4 × 10^−5^ mol/L rapamycin group on HTrF. (**A**-**e**): The number of migrated HTrF cells in the 6 × 10^−5^ mol/L rapamycin group on HTrF. (**A**-**f**): The number of migrated HTrF cells in the 8 × 10^−5^ mol/L rapamycin group on HTrF. (**A**-**g**): The number of migrated HTrF cells in the 10 × 10^−5^ mol/L rapamycin group on HTrF. (**B**-**a**): The number of migrated HTEpiC cells in the negative control group on HTEpiC. (**B**-**b**): The number of migrated HTEpiC cells in the 1 × 10^−5^ mol/L rapamycin group on HTEpiC. (**B**-**c**): The number of migrated HTEpiC cells in the 2 × 10^−5^ mol/L rapamycin group on HTEpiC. (**B**-**d**): The number of migrated HTEpiC cells in the 4 × 10^−5^ mol/L rapamycin group on HTEpiC. (**B**-**e**): The number of migrated HTEpiC cells in the 6 × 10^−5^ mol/L rapamycin group on HTEpiC. (**B**-**f**): The number of migrated HTEpiC cells in the 8 × 10^−5^ mol/L rapamycin group on HTEpiC. (**B**-**g**): The number of migrated HTEpiC cells in the 10 × 10^−5^ mol/L rapamycin group on HTEpiC.

The number of migrated HTrF cells in the negative control and 1 × 10^−5^, 2 × 10^−5^, 4 × 10^−5^, 6 × 10^−5^, 8 × 10^−5^, and 10 × 10^−5^ mol/L rapamycin concentration groups were (347.4 ± 6.73), (153.8 ± 6.09), (134.5 ± 4.91), (109.3 ± 2.60), (61.7 ± 1.51), (24.11 ± 3.77), and (17.56 ± 1.60), respectively. The number of migrated cells in each group was lower than that in the negative control group, and the differences were statistically significant, *p* < 0.05 (Figure 5A).

The number of migrated HTEpiC cells in the negative control and 1 × 10^−5^, 2 × 10^−5^, 4 × 10^−5^, 6 × 10^−5^, 8 × 10^−5^, and 10 × 10^−5^ mol/L rapamycin concentration groups were (206.0 ± 6.90), (158.5 ± 2.78), (138.2 ± 3.67), (120.7 ± 4.87), (65.3 ± 2.74), (60.0 ± 2.67), and (53.4 ± 2.81), respectively. The number of migrated cells in each group was lower than that in the negative control group, and the differences were statistically significant, *p* < 0.05 (Figure 5B).

### 3.4. Apoptosis Assay

**Figure 6:**
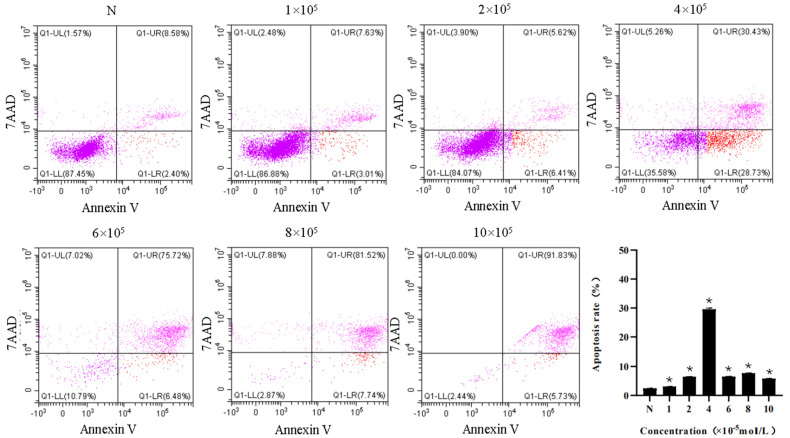
The apoptosis rates of HTrF with different concentrations of rapamycin. * represents a statistically significant difference compared to the negative control group, *p* < 0.05.

The apoptosis rates of HTrF in the negative control and 1 × 10^−5^, 2 × 10^−5^, 4 × 10^−5^, 6 × 10^−5^, 8 × 10^−5^, and 10 × 10^−5^ mol/L rapamycin concentration groups were (2.43 ± 0.04)%, (3.10 ± 0.03)%, (6.48 ± 0.04)%, (29.57 ± 0.43)%, (6.50 ± 0.12)%, (7.68 ± 0.11)%, and (5.83 ± 0.05)%, respectively. The apoptosis rate in each group was higher than that in the negative control group, and the differences were statistically significant (*p* < 0.05) (Figure 6).

## 4. Discussion

Restenosis after airway stenting in benign cicatricial airway stenosis (BCAS) seriously affects the prognosis of patients, making it an urgent problem to be solved in the field of respiratory intervention medicine [29,30]. Restenosis occurs due to the continuous stimulation of the stent aggravating the proliferation of cicatricial granulation tissue and the stent itself cannot inhibit the proliferation of cicatricial granulation tissue. In the past, our team has used an in-house manufactured paclitaxel-eluting stent to treat BCAS. We found that paclitaxel inhibited fibroblasts as well as epithelial cells, leading to the failure of airway epithelialization, and restenosis could not be treated [22,23]. CCK-8, wound healing, Transwell migration, and apoptosis assays were performed to detect the effects of rapamycin on the survival, proliferation, migration, and apoptosis of HTrF and HTEpiC. The results showed that compared to paclitaxel and mitomycin C, rapamycin significantly inhibited the proliferation and migration and promoted the apoptosis of HTrF, and had the least effect on HTEpiC. The optimal concentration range of rapamycin was 10–40 nM.

The results of the CCK-8 assay showed that paclitaxel, mitomycin C, and rapamycin at different concentrations and action times inhibited the proliferation and migration and promoted apoptosis of HTrF and HTEpiC to varying degrees. The effective concentrations of paclitaxel, mitomycin C, and rapamycin required to inhibit HTrF by more than 50% were 10^−7^–10^−4^ mol/L, 10^−6^–10^−4^ mol/L, and 10^−5^–10^−4^ mol/L, respectively. Although the effective concentration range of rapamycin was narrower than that of the paclitaxel and mitomycin C, within the effective range, its ability to inhibit HTrF proliferation was not different from that of the other two drugs. Within the effective concentration range, the inhibition rate of paclitaxel and mitomycin C on HTEpiC was greater than 50%, indicating that paclitaxel and mitomycin C significantly inhibited HTEpiC while inhibiting HTrF, making airway epithelial cells unable to cover the submucosa, which resulted in the failure of fibroblasts in the submucosa to be inhibited, leading to the unsatisfactory effect of reducing restenosis after airway stenting, consistent with the results from our previous studies [22,23]. However, the inhibition rate of rapamycin on HTEpiC was less than 20% within the effective concentration range, indicating that rapamycin not only inhibited the proliferation of fibroblasts, but also inhibited the epithelial cells to a minimum, achieving a dynamic balance between the two cells which could contribute to the epithelialization of the airway, and alleviate restenosis. It has been confirmed elsewhere that rapamycin can inhibit the proliferation of fibroblasts in the cicatricial tissue without significantly inhibiting bladder epithelial cells and vascular endothelial cells, resulting in the inhibition of restenosis after stenting. In the wound healing assay, there were no significant differences between the negative control and 10^−5^ mol/L rapamycin groups in terms of the healing rate of HTEpiC, probably because the doubling time of HTEpiC is greater and the migration speed is lower than HTrF. Moreover, the area measured using ImageJ software was not accurate, probably inducing a measurement error. We further improved the Transwell migration assay to verify the above results and to make up for the error introduced in the wound healing assay measurement. The apoptosis rates in the (1–4) × 10^−5^ mol/L rapamycin concentration groups were higher than that in the negative control group, and increased with the increase in rapamycin concentration, suggesting that rapamycin could promote the apoptosis of HTrF. However, the apoptosis rates in the (6–10) × 10^−5^ mol/L rapamycin concentration groups decreased compared to the (1–4) × 10^−5^ mol/concentration groups, because the number of dead cells increased significantly, resulting in fewer apoptotic cells.

The innovations of the current study are: firstly, similar to the successful use of rapamycin in inhibiting restenosis after stenting in other systems, we observed the effects of rapamycin in the field of respiratory intervention medicine; secondly, the cells used in the study were passaged for 1–5 generations, which is closer to the actual clinical situation, and therefore, the probability of the intervention being successful in the clinical setting is higher; thirdly, paclitaxel and mitomycin C, which are commonly used as coating drugs, were used as controls to detect the multi-dimensional characteristics of rapamycin using various methods, making the results more reliable. The current study also had certain limitations: the study was performed in vitro, and molecular biology experiments and animal experiments need to be performed to further verify the effectiveness and feasibility of rapamycin in inhibiting restenosis after airway stenting.

Despite these limitations, the study compared the effects of paclitaxel, mitomycin C, and rapamycin on HTrF and HTEpiC, screened rapamycin, which has the least effect on epithelialization, and determined its optimal concentration range. Rapamycin may become an ideal coating drug for airway stents, making the current study an experimental basis for further research and the improvement of drug-eluting stents. Molecular biology experiments and animal experiments need to be performed to clarify the effectiveness and feasibility of rapamycin. Our team will continue to improve the research methodology to provide a basis and direction for solving the problem of restenosis after airway stenting.

## 5. Conclusions

Compared to paclitaxel and mitomycin C, rapamycin not only effectively inhibited HTrF, but also had the least effect on HTEpiC. The optimal concentration range of rapamycin was (1–4) × 10^−5^ mol/L (i.e., 10–40 nM).

## Figures and Tables

**Table 1:** The inhibitory rates of paclitaxel, mitomycin C, and rapamycin on the proliferation of Human Tracheal Fibroblasts (HTrF).

Concentration (mol/L)	Paclitaxel			Mitomycin C			Rapamycin		
	24 h	48 h	72 h	24 h	48 h	72 h	24 h	48 h	72 h
10^−11^	2.62 ± 0 *	6.31 ± 1.05 *	8.63 ± 1.42 *	3.85 ± 1.29 *	5.15 ± 0.57 *	6.23 ± 1.92 *	1.08 ± 0.38 *	3.87 ± 0.47 *	7.94 ± 0.88 *
10^−10^	4.18 ± 0.17 *	11.70 ± 1.46 *	13.64 ± 1.05 *	16.26 ± 1.08 *	13.08 ± 1.20 *	16.42 ± 1.42 *	5.29 ± 0.85 *	6.90 ± 0.58 *	13.38 ± 0.71 *
10^−9^	10.24 ± 0.87 *	17.26 ± 1.32 *	18.81 ± 0.63 *	18.06 ± 1.34 *	17.93 ± 1.47 *	19.61 ± 1.45 *	12.09 ± 1.05 *	21.81 ± 2.66 *	26.37 ± 1.87 *
10^−8^	13.88 ± 1.67 *	34.25 ± 1.66 *	36.27 ± 1.25 *	20.75 ± 1.07 *	19.59 ± 1.24 *	21.46 ± 2.33 *	17.10 ± 1.03 *	33.32 ± 1.82 *	35.92 ± 1.10 *
10^−7^	46.15 ± 1.78 *	57.76 ± 0.54 *	57.91 ± 1.60 *	23.30 ± 0.80 *	25.85 ± 1.02 *	30.81 ± 1.56 *	19.87 ± 0.89 *	39.59 ± 0.45 *	42.87 ± 3.41 *
10^−6^	50.78 ± 1.77 *	62.06 ± 0.64 *	62.05 ± 1.77 *	38.87 ± 1.04 *	42.39 ± 0.73 *	51.31 ± 1.73 *	21.61 ± 0.99 *	40.27 ± 0.40 *	46.82 ± 1.00 *
10^−5^	55.75 ± 1.77 *	64.40 ± 1.05 *	65.99 ± 1.04 *	64.82 ± 1.16 *	78.02 ± 1.47 *	91.34 ± 1.18 *	27.76 ± 1.03 *	49.17 ± 0.94 *	69.00 ± 0.97 *
10^−4^	92.92 ± 0.55 *	96.06 ± 0.26 *	96.36 ± 0.24 *	94.37 ± 0.43 *	98.23 ± 0.16 *	98.18 ± 0.37 *	92.37 ± 0.56 *	96.30 ± 0.26 *	97.36 ± 0.22 *

* represents a statistically significant difference compared to the negative control group, *p* < 0.05.

**Table 2:** The inhibitory rates of paclitaxel, mitomycin C, and rapamycin on the proliferation of Human Tracheal Epithelial Cells (HTEpiC).

Concentration (mol/L)	Paclitaxel			Mitomycin C			Rapamycin		
	24 h	48 h	72 h	24 h	48 h	72 h	24 h	48 h	72 h
10^−11^	7.59 ± 1.29 *	11.09 ± 0.52 *	13.99 ± 1.32 *	2.15 ± 0.12 *	2.49 ± 0.10 *	4.62 ± 0.82 *	1.75 ± 0.38 *	2.02 ± 0.48 *	4.07 ± 0.89 *
10^−10^	11.89 ± 1.29 *	14.56 ± 1.19 *	18.04 ± 1.28 *	4.87 ± 0.68 *	5.10 ± 0.56 *	9.65 ± 1.30 *	2.26 ± 0.96 *	3.25 ± 0.56 *	4.97 ± 1.57 *
10^−9^	14.48 ± 1.54 *	22.86 ± 1.31 *	25.51 ± 1.24 *	6.02 ± 1.11 *	6.33 ± 1.12 *	10.69 ± 1.22 *	3.47 ± 0.45 *	3.99 ± 1.24 *	6.03 ± 0.64 *
10^−8^	25.40 ± 0.57 *	27.76 ± 1.82 *	28.68 ± 1.63 *	6.91 ± 0.58 *	7.56 ± 1.41 *	16.92 ± 0.06 *	3.89 ± 0.76 *	5.54 ± 0.71 *	6.49 ± 0.77 *
10^−7^	33.82 ± 0.54 *	32.04 ± 2.11 *	43.03 ± 1.12 *	10.32 ± 0.49 *	15.54 ± 0.65 *	52.91 ± 1.08 *	3.95 ± 0.98 *	5.88 ± 0.91 *	9.22 ± 0.33 *
10^−6^	34.21 ± 0.91 *	41.24 ± 1.14 *	49.49 ± 0.86 *	45.59 ± 1.25 *	62.15 ± 1.05 *	88.11 ± 0.69 *	5.37 ± 1.02 *	6.17 ± 1.35 *	9.40 ± 0.60 *
10^−5^	35.96 ± 1.30 *	49.75 ± 1.74 *	55.22 ± 1.43 *	78.41 ± 0.75 *	89.25 ± 0.37 *	93.82 ± 0.96 *	7.25 ± 1.54 *	8.28 ± 0.47 *	10.19 ± 0.35 *
10^−4^	88.96 ± 0.49 *	91.05 ± 0.45 *	93.19 ± 0.45 *	92.01 ± 0.30 *	93.89 ± 0.68 *	94.94 ± 0.54 *	81.29 ± 1.06 *	94.41 ± 0.87 *	94.55 ± 0.71 *

* represents a statistically significant difference compared to the negative control group, *p* < 0.05.

## Data Availability

Not applicable.
